# The Stress and Adversity Inventory for Adults (Adult STRAIN) in Brazilian Portuguese: Initial Validation and Links With Executive Function, Sleep, and Mental and Physical Health

**DOI:** 10.3389/fpsyg.2019.03083

**Published:** 2020-01-28

**Authors:** Milton J. Cazassa, Margareth da S. Oliveira, Chandler M. Spahr, Grant S. Shields, George M. Slavich

**Affiliations:** ^1^Department of Psychology, Pontifical Catholic University of Rio Grande do Sul, Porto Alegre, Brazil; ^2^Department of Psychology, San Diego State University, San Diego, CA, United States; ^3^Center for Mind and Brain, University of California, Davis, Davis, CA, United States; ^4^Cousins Center for Psychoneuroimmunology and Department of Psychiatry and Biobehavioral Sciences, University of California, Los Angeles, Los Angeles, CA, United States

**Keywords:** life stress, early adversity, assessment, measurement, allostatic load, risk, health, disease

## Abstract

It has been widely hypothesized that stressors occurring over the lifespan exert a cumulative impact on health, but little work has directly tested these theories given the difficulty associated with measuring cumulative stress exposure over the lifespan. We addressed this issue in Brazil by translating the Stress and Adversity Inventory for Adults (Adult STRAIN) into Brazilian Portuguese. We then examined the instrument’s usability and acceptability; concurrent, discriminant, predictive, and incremental validity; and test–retest reliability. Participants were 330 Brazilian adults (238 women; *M*_age_ = 32.16; range: 18–76 years old) who completed the Adult STRAIN in Brazilian Portuguese, Childhood Trauma Questionnaire-Short Form (CTQ-SF), and Perceived Stress Scale (PSS). They also completed measures of socioeconomic status, personality, social desirability, negative affect, physical and mental health complaints, sleep quality, executive function, and doctor-diagnosed general health problems and autoimmune disorders. The STRAIN exhibited excellent usability and acceptability and was completed in 16 min and 27 s, on average. It showed good concurrent validity relative to the CTQ-SF and PSS (*rs* ≥ 0.377) and good discriminant validity, both with and without adjusting for covariates. In addition, the STRAIN significantly predicted all of the health outcomes assessed except for executive function and explained substantial variance in these outcomes over and above the CTQ-SF, PSS, and covariates assessed. Finally, the test–retest reliability indices for total lifetime stressor count and severity were outstanding (*r*_icc_ = 0.936 and 0.953, respectively, over *M* = 34.86 days). The Adult STRAIN in Brazilian Portuguese thus exhibits excellent usability and acceptability, good concurrent and discriminant validity, strong predictive and incremental validity across a variety of health outcomes, and outstanding test–retest reliability. We therefore conclude that the STRAIN is a practical, valid, and reliable instrument for researchers and clinicians looking to efficiently assess cumulative lifetime stress exposure in Brazilian Portuguese.

## Introduction

A large literature demonstrates that acute and chronic stress exposure can affect individuals’ quality of life, subjective wellbeing, and mental and physical health (Sadir et al., 2010; Slavich, 2016a, b; Epel et al., 2018). Depending on their frequency, intensity, type, and duration, life stressors can alter psychological, neural, endocrine, and inflammatory processes that in turn promote risk for a number of disorders, including anxiety disorders, depression, asthma, metabolic syndrome, heart disease, certain types of cancer, neurodegeneration, and cognitive decline (Slavich et al., 2010; Slavich and Irwin, 2014). Assessing life stress exposure is thus critical for understanding key factors affecting disease risk and longevity (Shields and Slavich, 2017).

Although many studies have assessed recent life stress exposure in relation to health, very few have taken a life course perspective and assessed all of the acute and chronic stressors that individuals have experienced over the entire lifespan (Malat et al., 2017). This has occurred despite the fact that most contemporary models of stress and health posit that stress can exert cumulative effects on health and wellbeing, whereby stress burden is hypothesized to accumulate over time and eventually lead to the emergence of disease (e.g., McEwen, 1998; Graham et al., 2006; Lupien et al., 2009). A primary reason for this lack of research involves the difficulty associated with assessing lifetime stress exposure in a time- and cost-efficient manner (Slavich, 2019). Recently, however, George M. Slavich developed an online system for measuring stress called the Stress and Adversity Inventory (STRAIN), which efficiently assesses all of the acute life events and chronic difficulties that individuals have experienced over the lifespan. One version of the STRAIN is designed specifically for adolescents (i.e., Adolescent STRAIN) (Slavich et al., 2019a) and a second version is designed for adults (i.e., Adult STRAIN) (Slavich and Shields, 2018).

Both versions of the STRAIN have demonstrated good usability and acceptability, and very good concurrent and discriminant validity (Slavich and Shields, 2018; Slavich et al., 2019a). Moreover, the STRAIN has shown excellent test–retest reliability over 2–4 weeks (*r*s = 0.904 –0.919) (Slavich and Shields, 2018) in addition to consistent predictive validity in relation to a variety of health-related outcomes. These outcomes include memory and decision making (Goldfarb et al., 2017; Shields et al., 2017a), executive function (Slavich and Shields, 2018), working memory capacity (Shields et al., 2019a), diurnal cortisol levels (Cuneo et al., 2017), biological responses to acute stress (Lam et al., 2019), metabolic function (Kurtzman et al., 2012), biological aging (Mayer et al., 2019), fatigue and depression (Bower et al., 2014; Dooley et al., 2017; Pegg et al., 2019), birth timing (Gillespie et al., 2017), prenatal health behaviors (Smith et al., in press), sleep problems (Slavich and Shields, 2018), suicidal behavior (Stewart et al., 2019), hypertension and diabetes risk (Olvera Alvarez et al., 2019), self-reported physical and mental health complaints (Toussaint et al., 2016; Shields et al., 2017b), and doctor-diagnosed physical illnesses and autoimmune disorders (Slavich and Shields, 2018; see also Slavich and Toussaint, 2014; Schüssler-Fiorenza Rose et al., 2019). To date, however, the STRAIN has been validated only in English (Slavich and Shields, 2018; Slavich et al., 2019a) and German (Sturmbauer et al., 2019), thus limiting its usability and potential impact.

To address this issue, we conducted a formal translation of the Adult STRAIN into Brazilian Portuguese. Then, we examined the usability, acceptability, and concurrent, predictive, and incremental validity of the Adult STRAIN in Brazilian Portuguese by following the same validation protocol used for the English version (see Slavich and Shields, 2018). Based on the research reviewed above, we hypothesized that the Adult STRAIN in Brazilian Portuguese would demonstrate good usability and acceptability and be significantly correlated with other life stress measures. Furthermore, we hypothesized that cumulative lifetime stress exposure, as measured by the Brazilian Portuguese STRAIN, would be associated with the six outcomes assessed but that these effects would vary by stressor type, which has been found previously with the Adult STRAIN (Slavich and Shields, 2018; Slavich et al., 2019a; Sturmbauer et al., 2019) and with other interview-based measures of life stress (e.g., the Life Events and Difficulties Schedule; Brown et al., 1995).

## Materials and Methods

### Participants and Procedure

Out of the 510 participants who began this online study, 139 (27.3%) failed at least one of the five attention checks that were designed to ensure high-quality data. These attention checks were interspersed throughout the study measures and response options (e.g., “Check this box to show that you are paying attention,” “Please answer *yes* to this question to show that you are paying attention”). In addition, 35 participants (6.9%) began the STRAIN but discontinued at some point during the protocol. An additional six individuals were excluded prior to starting the study because they did not meet the minimum age requirement (at least 18 years old; *n* = 2) or education requirement (at least a secondary education; *n* = 4). The final sample thus consisted of 330 participants (238 women, 92 men) with a mean age of 32.16 years old (*SD* = 13.55) and substantial variability across the adult lifespan (range: 18–76 years old).

The sample was diverse in terms of socioeconomic status. According to Brazilian standards (Associação Brasileira de Empresas de Pesquisa [ABEP], 2016; Kamakura and Mazzon, 2016), for example, 70 participants were classified as being in class A (i.e., high class), 73 as B1 (i.e., high middle class), 84 as B2 (i.e., average middle class), 54 as C1 (i.e., low middle class), and 49 as C2/D/E (i.e., low class). Regarding race, most of the sample was white (*n* = 290), with fewer participants self-identifying as black/mixed-race (*n* = 32) or other (Asian and people who did not want to report race; *n* = 8). Finally, regarding religion, most of the sample self-identified as Catholic (*n* = 137), but many also identified as being Spiritists (*n* = 40), Spiritualists without religion (*n* = 63), atheists (*n* = 42), or other (Umbanda, Evangelical, Protestant, Adventist, Buddhist, and others; *n* = 33) (see Table 1).

**TABLE 1:** Lifetime stressor count and lifetime stressor severity according to participants’ demographic characteristics.

**Participant**	**Sample**	**Lifetime stressor**	**Lifetime stressor**
**characteristics**	***n***	**count *M* (*SD*)^a^**	**severity *M* (*SD*)^a^**
**Gender**			
Male	91	20.67 (12.69)	49.84 (30.87)
Female	238	23.35 (13.49)	60.43 (32.77)
Transgender	1	24.00 (−)	65.00 (−)
**Age and Gender**			
18–29 years old	188	21.03 (11.38)	52.88 (27.58)
Male	53	18.79 (11.27)	46.30 (28.10)
Female	134	21.90 (11.38)	55.40 (27.13)
Transgender	1	24.0 (−)	65.0 (−)
30–39 years old	61	24.46 (13.95)	62.62 (33.77)
Male	19	26.74 (13.41)	63.47 (34.25)
Female	42	23.43 (14.22)	62.24 (33.96)
40–49 years old	27	22.22 (14.84)	59.56 (38.92)
Male	5	9.20 (9.98)	22.40 (25.17)
Female	22	25.18 (14.29)	68.0 (36.76)
50–59 years old	37	27.92 (18.37)	70.89 (43.42)
Male	9	25.89 (14.98)	60.11 (34.74)
Female	28	28.57 (19.53)	74.36 (45.88)
60+ years old	17	22.59 (12.82)	58.24 (33.01)
Male	5	19.60 (12.95)	44.40 (24.23)
Female	12	23.83 (13.12)	64.00 (35.33)
**Socioeconomic Status^b^**			
A	70	18.20 (11.09)	48.54 (28.51)
B1	73	21.22 (11.18)	53.59 (28.71)
B2	84	23.04 (11.41)	59.25 (29.48)
C1	54	24.94 (15.00)	61.63 (34.72)
C2 + D + E	49	27.71 (17.56)	68.15 (41.59)
**Religion^c^**			
Catholic	137	19.22 (11.95)	48.85 (29.64)
Spiritist	40	26.83 (13.00)	68.50 (30.48)
Spiritualist without religion	63	25.38 (13.93)	63.16 (33.16)
Atheist	42	21.50 (10.26)	55.86 (25.50)
Other	33	27.06 (18.27)	67.97 (45.28)
**Race^d^**			
White	290	21.39 (12.00)	54.36 (29.31)
Black/Mixed race	32	32.84 (18.99)	81.97 (44.16)
Other	8	26.25 (14.18)	74.63 (45.70)

*^*a*^*M* = mean; *SD* = standard deviation; ^*b*^socioeconomic status A = high class, B1 = high middle class, B2 = average middle class, C1 = low middle class, C2/D/E = low class; ^*c*^religion others = Umbanda, Evangelical, Protestant, Adventist, Buddhist, and others; ^*d*^race others = Asian and people who did not want to report. Results did not differ when the individual who identified as transgender was removed from analyses.*

Participants were recruited using print and social media advertisements posted widely in community locations. Written informed consent was first obtained; then, a copy of the signed consent form was emailed to the participant. Participants could start and stop the study protocol at any time, and when they completed all of the measures, they were thanked for their time. All procedures were approved by the relevant Brazilian research bodies (i.e., Scientific Commission and the Ethics Committee in Research from the Pontifical Catholic University of Rio Grande do Sul) and adhered to Brazilian Resolution 466 of December 12, 2012, of the National Health Council of Brazil, Ministry of Health (CNS 46/12).

## Life Stress Measures

### Stress and Adversity Inventory (STRAIN)

A seven-step procedure was used to translate the Adult STRAIN from English into Brazilian Portuguese. First, two translators who were fluent in both English and Brazilian Portuguese independently translated the Adult STRAIN into Brazilian Portuguese. Second, three independent, bilingual experts evaluated both versions of the interview and noted possible translation issues. Third, the translation issues were checked and addressed, and the revised version of the Adult STRAIN in Brazilian Portuguese was then evaluated by a second set of two independent experts. Fourth, following revisions, a focus group of psychologists and psychology graduate students assessed the comprehensibility of the newly translated version. Fifth, a bilingual expert reviewed the final translated version of the Adult STRAIN and compared it with both the original version and the back-translated version. Sixth, based on this review, final adjustments were made to the Adult STRAIN in Brazilian Portuguese. Lastly, a pilot study was conducted to finalize the user interface and to ensure the basic usability of the platform.

**FIGURE 1:**
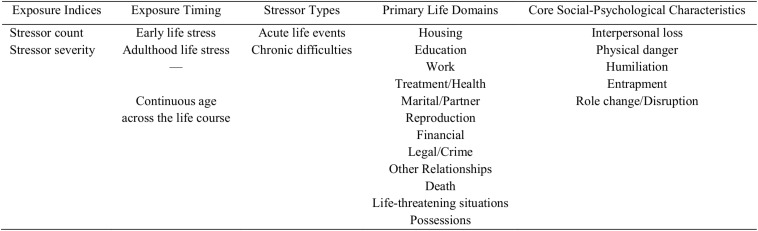
Dimensions of life stress assessed by the Stress and Adversity Inventory for Adults (Adult STRAIN).

Like the original Adult STRAIN in English, which has been previously described in detail (Slavich and Shields, 2018), the Adult STRAIN in Brazilian Portuguese assesses individuals’ exposure to 55 different stressors across the lifespan, including 26 acute life events and 29 chronic difficulties (see https://www.STRAINsetup.com). As summarized in Figure 1 (Slavich and Shields, 2018), these stressors span two stress exposure indices, two stress exposure timing categories, two stressor types, 12 primary life domains, and five core social-psychological characteristics. If a participant reports having experienced a particular stressor, follow-up questions are prompted to determine the stressor’s severity, frequency, timing, and duration. Based on participants’ answers, hundreds of raw variables are generated, which can then be combined to create more than 115 summary scores that provide a comprehensive snapshot of individuals’ lifetime stress exposure in its various forms. In the present study, we focused mainly on the two primary stress exposure indices – namely, total lifetime stressor count, indexed as the total number of life stressors a person experienced, and total lifetime stressor severity, indexed as the cumulative severity of all of the stressors a person experienced. This approach replicated the procedure followed in the original Adult STRAIN validation study (i.e., Slavich and Shields, 2018).

### Childhood Trauma Questionnaire-Short Form (CTQ-SF)

Early adversity was assessed using the Childhood Trauma Questionnaire-Short Form (CTQ-SF), which is one of the most widely used instruments for measuring childhood abuse and neglect. The 28-item CTQ-SF is a retrospective, self-report questionnaire for adolescents and adults that assesses physical abuse, emotional abuse, sexual abuse, physical neglect, and emotional neglect. Responses are provided using a Likert scale, ranging from 1 (*Never*) to 5 (*Always*), with higher scores representing greater adversity. The CTQ-SF predicts a variety of negative health outcomes, including substance abuse and related psychopathologies (Bernstein et al., 2003). The Brazilian version of the CTQ-SF was obtained from Grassi-Oliveira et al. (2006). We selected the 28-item version that was used in the original English STRAIN validation study, which has shown internal consistency ranging from satisfactory to excellent (α = 0.61–0.95) (Grassi-Oliveira et al., 2006). In the present sample, the overall internal consistency of the CTQ-SF was good (α = 0.82).

### Perceived Stress Scale (PSS)

A 10-item version of the Perceived Stress Scale (PSS) in Brazilian Portuguese (Reis et al., 2010; Machado et al., 2014; Faro, 2015), which corresponds to the original English version (Cohen et al., 1983), was used to measure participants’ perceived stress levels over the past month. The PSS is arguably the most widely used instrument for assessing stress. It focuses on how respondents view the uncontrollable and stressful aspects of their lives. Answers are provided on a Likert scale, ranging from 0 (*never*) to 4 (*always*), with higher scores representing greater perceived stress. The Brazilian Portuguese version of the PSS has shown evidence of validity and adequate internal consistency (α = 0.80) (Faro, 2015; see also Machado et al., 2014). In the present sample, the internal consistency of the PSS was excellent (α = 0.92).

### Socioeconomic and Potential Confounding Factors

#### Socioeconomic Status

Participants self-reported their age, gender, educational level, and monthly income, which were then used to establish their socioeconomic status according to establish criteria in Brazil (Andrade et al., 2010; Associação Brasileira de Empresas de Pesquisa [ABEP], 2016; Kamakura and Mazzon, 2016).

#### Big Five Personality Traits

Personality traits were assessed using the Ten Item Personality Inventory (TIPI), which measures extraversion, agreeableness, conscientiousness, neuroticism, and openness to experience (Hutz et al., 1998). In the United States and United Kingdom, the TIPI has been shown to exhibit adequate convergent validity and significant associations with longer assessments of the Big Five personality traits, with reliability and test–retest indices that are comparable to the Big Five Inventory (Gosling et al., 2003; Woods and Hampson, 2005). In the present study, we used the Brazilian Portuguese version of the TIPI, which has been shown to exhibit the expected factor structure and to have good convergent validity with the Big Five Inventory, good 4-week test–retest reliability (*r*s > 0.71), and to predict self-esteem, affect, and aggressiveness (Nunes et al., 2018). Given the relatively brief nature of the TIPI and its emphasis on high content validity, the internal consistency of the TIPI in this sample was low for several of the Big Five traits (Extraversion *r*_SB_ = 0.685; Agreeableness *r*_SB_ = 0.243; Conscientiousness *r*_SB_ = 0.466; Neuroticism *r*_SB_ = 0.685; Openness to Experience *r*_SB_ = 0.399), which has been described as normal by the instrument’s authors (Gosling et al., 2003).

#### Social Desirability

The inclination to wish to make a good impression and to report socially desirable behaviors while omitting socially undesirable ones was assessed using Stöber’s 17-item Social Desirability Scale (SDS-17; Stöber, 2001). The scale includes true or false response options, and users’ answers are summed according to pre-defined criteria to create an overall index of social desirability. The SDS-17 has shown excellent internal consistency (α = 0.94) and convergence with other social desirability scales (Stöber, 2001). It has also shown good validity in other countries, including Austria, Canada, and the United States (Tran et al., 2012). We found no studies that previously used this scale in Brazil. Therefore, following authorization from the instrument’s author, we translated the scale using a bilingual speaker (English and Portuguese) and then back-translated the instrument using another bilingual speaker. The back-translated version was then evaluated by a native English speaker and psychological scientist. After evaluating the back-translation, final adjustments were made to the translated scale. In the present sample, the internal consistency of the newly translated SDS-17 was acceptable (α = 0.72).

#### Negative Affect

The Positive and Negative Affect Schedule (PNAS) was used to assess participants’ levels of negative affect over the past week (Watson et al., 1988). This 20-item questionnaire measures 10 positive and 10 negative emotions. Responses are provided using a Likert scale ranging from 1 (*Not at all*) to 5 (*Very much*). The 10 questions assessing negative affect were averaged to form an overall negative affect index, with higher scores representing greater negative affect. The Zanon and Hutz (2014) Brazilian version of the PANAS was used in this study. In the present sample, the internal consistency of the negative affect scale used in analyses was very good (α = 0.85).

### Cognitive Measures

#### Executive Function

Executive functioning was measured using a version of the Stroop task programed in jsPsych (De Leeuw, 2015), which was used and described in detail in the original Adult STRAIN validation study (see Slavich and Shields, 2018). The task was translated into Brazilian Portuguese for the present purposes. The translation procedure for this task was the same as the translation procedure for the SDS-17 (see above). Due to technical difficulties, nine participants were unable to complete the task. We observed the classic Stroop effect in this study, with interference from incongruent (vs. congruent) words slowing reporting of word color by an average of 122.42 ms, *t*(317) = 32.36, *p* < 0.001. Because Stroop effects represent the extent to which goal-irrelevant information interferes with reporting of goal-relevant information (and thus inefficient inhibitory control of cognition), higher scores indicate poorer executive function.

### Health Measures

#### Sleep Quality

The Brazilian Portuguese version of the Pittsburgh Sleep Quality Index (PSQI-BR; Bertolazi, 2008) was used to measure participants’ sleep quality. This instrument was developed by Buysse et al. (1989) and is one of the most widely used instruments for assessing objective and subjective aspects of sleep quality. The PSQI-BR includes self-administered questions and other questions to be answered by a roommate (Bertolazi, 2008). In the present study, we only used the self-administered questions in order to match the original STRAIN validation study (Slavich and Shields, 2018). In the present sample, the internal consistency of the instrument was acceptable (α = 0.72).

#### General Mental Health Complaints

Participants’ number of general mental health complaints over the past month was assessed using the Kessler-6 Psychological Distress Inventory (K-6), which is a brief six-item scale that measures non-specific psychological distress (i.e., as opposed to disorder-specific psychiatric diagnoses). The scale demonstrates excellent internal consistency (α = 0.91) (Kessler et al., 2002) and possesses satisfactory sensitivity for predicting severe mental illness (Kessler et al., 2003, 2010). Answers are provided on a Likert scale ranging from 1 (*never*) to 5 (*the whole time*), with higher scores representing a greater number of mental health complaints. The Brazilian Portuguese translation of the scale was obtained through the instrument’s website (Kessler, 2008). In the present sample, the internal consistency of the instrument was very good (α = 0.89).

#### General Physical Health Complaints

Participants’ number of general physical health complaints was assessed using the Physical Health Questionnaire (PHQ). This 14-item scale assesses a variety of physical and somatic symptoms experienced over the past month, with good construct validity (Schat et al., 2005). Responses indicating symptom frequency were summed together to create an overall physical health problem index, with higher scores representing a greater number of general physical health complaints. Since we found no studies that used this scale in Brazil, following authorization from the instrument’s author, we translated the scale using the same procedure that we used for translating the SDS-17 and Stroop task to create the PHQ-BR. In the present sample, the internal consistency of this new PHQ-BR was good (α = 0.82).

#### Doctor-Diagnosed General Health Problems

Number of general health problems diagnosed by a physician was measured using the question “Have you ever been diagnosed by a doctor with any of the following conditions? (Check all that apply),” written in Brazilian Portuguese. The following conditions were included: anxiety, arthritis (not-rheumatoid or psoriatic), asthma, cancer, chronic pain, heart disease, depression, gastroesophageal reflex disease (or chronic heartburn), heart attack, high blood pressure, insomnia, kidney stone, migraine, overweight, posttraumatic stress disorder, stomach ulcer, and stroke. Responses were summed to create an overall physical health problem index, with higher scores representing a greater number of physician-diagnosed general health problems.

#### Doctor-Diagnosed Autoimmune Disorders

Number of autoimmune diseases diagnosed by a physician was measured using the question “Have you ever been diagnosed by a doctor with any of the following conditions? (Check all that apply),” written in Brazilian Portuguese. The following conditions were included: Addison’s disease (primary adrenal insufficiency), celiac disease (gluten intolerance), dermatomyositis, Grave’s disease (hyperthyroidism), Hashimoto thyroiditis (inflammation of the thyroid), inflammatory bowel disease (i.e., Crohn’s disease, ulcerative colitis), multiple sclerosis, myasthenia gravis, pernicious anemia, psoriasis (or psoriatic arthritis), rheumatoid arthritis, Sjögren’s syndrome (autoimmune disease characterized by dry eyes and dry mouth), lupus (systemic lupus erythematosus), and other autoimmune disorder (specify). Responses to this last option were examined and included if they represented a known autoimmune disorder. Then, all responses were summed to create a general autoimmune disorder index, with higher scores representing a greater number of physician-diagnosed autoimmune disorders.

### Data Analyses

Data were processed and analyzed using SPSS version 20.0. For descriptive analyses, the Pearson parametric correlation analyses were conducted for continuous variables (i.e., age) and the ANOVA (*Post hoc* Tukey) technique was used for categorical variables (i.e., socioeconomic status, religion, race, gender, and educational level). To calculate effect sizes, we used Partial Eta-Squared for categorical variables and the determination coefficient (Cohen’s *f*_2_) for continuous variables. Furthermore, Pearson parametric correlation analyses were conducted to verify the concurrent validity of STRAIN with the PSS and CTQ-SF. To assess discriminant validity, Pearson parametric correlations were used to analyze associations between the two main STRAIN outcomes (i.e., lifetime stressor count and lifetime stressor severity), as well the PSS and CTQ-SF, with the SDS-17 and the TIPI. Discriminant validity was also examined using a linear regression model to verify these associations while controlling for covariates (i.e., gender, age, socioeconomic status, race, and negative affect). Predictive validity was examined using Pearson parametric correlations and multiple linear regression models for mental (K-6) and physical (PHQ-BR) health complaints, sleep quality (PSQI-BR), and executive function (Stroop), whereas Poisson generalized linear models were applied to examine doctor-diagnosed general health problems and autoimmune disorders. To examine the STRAIN’s comparative predictive validity, we used multiple linear regression analyses for the continuous health outcomes (i.e., PHQ-BR, K-6, PSQI-BR, and Stroop), and Poisson regression analyses for doctor-diagnosed general health problems and autoimmune disorders. Test-rest reliability was examined using intraclass correlation coefficients (*r*_icc_) and Pearson correlations. For examining differences in lifetime stressor exposure for men and women, we used an independent samples *t*-test. Finally, we used Poisson generalized linear model for examining the STRAIN’s predictive validity in relation to participants’ probability of being diagnosed with an autoimmune disorder according to stressor timing, type, primary life domain, and core social-psychological characteristic. Effect size measures (e.g., Cohen’s *d*, β, *r*, IRR) are reported when relevant.

## Results

### Usability and Acceptability of the STRAIN

A total of 371 participants passed all of the attention checks and 35 began the STRAIN but discontinued prior to completion, producing a high completion rate of 93.1%. No participants reported any problems with the system or emotional discomfort or complaints following the interview. The mean time to complete the STRAIN was 16 min and 27 s (interquartile range = 10 min, 6 s – 18 min and 25 s).

### Descriptive Statistics

Participants experienced an average of 22.62 stressors over the life course (*SD* = 13.29; range 0–81; possible range 0–166), with a mean total lifetime stressor severity of 57.52 (*SD* = 32.51; range: 0–177; possible range 0–265). These two stress exposure indices were strongly inter-correlated (*r* = 0.935, *p* < 0.001). In addition, total number of lifetime stressors assessed by the STRAIN was significantly associated with participants’ age (*r* = 0.129, *p* = 0.019; *f*_2_ = 0.0169), socioeconomic status (*F*(4,325) = 4.558, *p* = 0.001; η_*p*_^2^ = 0.053), religion (*F*(4,310) = 5.071, *p* = 0.001; η_*p*_^2^ = 0.062), and race (*F*(2,327) = 11.728, *p* < 0.001; η_*p*_^2^ = 0.067), but not with gender (*p* = 0.261). Total lifetime stressor severity, in turn, was associated with age (*r* = 0.151, *p* = 0.006; *f*_2_ = 0.0233), gender (*F*(2,327) = 3.578, *p* = 0.029; η_*p*_^2^ = 0.021), socioeconomic status (*F*(4,325) = 3.217, *p* = 0.013; η_*p*_^2^ = 0.038), religion (*F*(4,310) = 5.095, *p* = 0.001; η_*p*_^2^ = 0.062), and race (*F*(2,327) = 12.321, *p* < 0.001; η_*p*_^2^ = 0.070). Neither total lifetime stressor count nor total lifetime stressor severity were related to participants’ educational level (*F*(3,326) = 1.246, *p* = 0.293, and *F*(3,326) = 0.228, *p* = 0.877, respectively).

Table 1 describes the number of participants in the sample separated by gender, age group, socioeconomic status, religion, and race. Also displayed are the means and standard deviations obtained for lifetime stressor count and lifetime stressor severity across these groupings. Consistent with the general pattern of results obtained for the English Adult STRAIN (Slavich and Shields, 2018), a greater number of lifetime stressors was experienced by women (albeit not significantly so in this sample), older individuals, and socioeconomically disadvantaged groups. The same pattern was also evident for lifetime stressor severity. In contrast with the English validation study, in the present sample, blacks and mixed-race experienced significantly more stressors and greater lifetime stressor severity as compared to those self-identifying as white (*p* values < 0.001). In addition, religion was significantly associated with participants’ lifetime stress exposure, with Catholics reporting fewer lifetime stressors and less lifetime stressor severity as compared to those self-identifying with other religions or as atheist (*p* values < 0.029).

### Concurrent Validity

Following the same pattern of results obtained in the original Adult STRAIN validation study (Slavich and Shields, 2018), lifetime stressor count, as assessed by the Brazilian version of the Adult STRAIN, was significantly correlated with other measures of both early adversity (i.e., CTQ-SF: *r* = 0.594, *p* < 0.001) and perceived stress (i.e., PSS: *r* = 0.377, *p* < 0.001). Similar effects were found for lifetime stressor severity as assessed by the STRAIN, whereby the STRAIN was again significantly correlated with both the CTQ-SF (*r* = 0.579, *p* < 0.001) and PSS (*r* = 0.393, *p* < 0.001). These results provide evidence of the concurrent validity of Adult STRAIN in Brazilian Portuguese.

### Discriminant Validity

Next, we evaluated the discriminant validity of the STRAIN – first for lifetime stressor count and second for lifetime stressor severity – and then compared it to the discriminant validity of the CTQ-SF and PSS. Because the STRAIN was developed to assess life stress exposure (e.g., as opposed to emotional response), we expected that it would not be strongly related to participants’ social desirability or personality characteristics, as we have previously observed (Slavich and Shields, 2018). Contrary to expectations, lifetime stressor count and lifetime stressor severity were weakly but significantly related to social desirability (*r* = −0.209, *p* < 0.001, and *r* = −0.208, *p* < 0.001, respectively). Adjusting for covariates (i.e., gender, age, socioeconomic status, race, and negative affect) did not alter these associations (lifetime stressor count: β = −0.211, *p* < 0.001; lifetime stressor severity: β = −0.215, *p* < 0.001). The CTQ-SF (*r* = −0.232, *p* < 0.001) and PSS (*r* = −0.374, *p* < 0.001) were also significantly related to social desirability as assessed by the SDS-17, and these effects also remained significant while adjusting for covariates (i.e., gender, age, socioeconomic status, race, and negative affect) (CTQ: β = −0.179, *p* = 0.001; PSS: β = −0.186, *p* < 0.001).

With respect to personality, lifetime stressor count as assessed by the STRAIN was very weakly related to four personality factors assessed by the TIPI, namely: extraversion (*r* = −0.117, *p* = 0.033), agreeableness (*r* = −0.223, *p* < 0.001), conscientiousness (*r* = −0.109, *p* = 0.047), and neuroticism (*r* = 0.264, *p* < 0.001). When controlling for gender, age, socioeconomic status, race, and negative affect, however, lifetime stressor count was no longer related to extraversion (β = −0.036, *p* = 0.488) or conscientiousness (β = −0.021, *p* = 0.687), but the STRAIN was still related to agreeableness (β = −0.128, *p* = 0.014) and neuroticism (β = 0.135, *p* = 0.023), and became related to openness to experience (β = −0.144, *p* = 0.004). Lifetime stressor severity was also weakly but significantly related to the four personality factors assessed by the TIPI, namely: extraversion (*r* = −0.119, *p* = 0.031), agreeableness (*r* = −0.177, *p* = 0.001), neuroticism (*r* = 0.254, *p* < 0.001), and openness to experience (*r* = 0.111, *p* = 0.044). When controlling for gender, age, socioeconomic status, race, and negative affect, however, lifetime stressor severity was no longer significantly related to extraversion (β = −0.035, *p* = 0.486), agreeableness (β = −0.081, *p* = 0.114), or neuroticism (β = 0.113, *p* = 0.051), but the STRAIN was still related to openness to experience (β = 0.173, *p* = 0.001).

Similarly, the CTQ-SF was significantly correlated with three out of the five personality factors assessed by the TIPI, namely: extraversion (*r* = −0.198, *p* < 0.001), agreeableness (*r* = −0.255, *p* < 0.001), and neuroticism (*r* = 0.264, *p* < 0.001). The PSS, in turn, was significantly associated with all five personality factors assessed by the TIPI: extraversion (*r* = −0.177, *p* = 0.001), agreeableness (*r* = −0.249, *p* < 0.001), conscientiousness (*r* = −0.244, *p* < 0.001), neuroticism (*r* = 0.566, *p* < 0.001), and openness to experience (*r* = −0.180, *p* = 0.001). When controlling for participants’ gender, age, socioeconomic status, race, and negative affect, the CTQ-SF was no longer related to neuroticism (β = 0.093, *p* = 0.123); however, it remained significantly related to two of the five personality factors (i.e., extraversion, β = −0.131, *p* = 0.013, and agreeableness, β = −0.171, *p* = 0.001), and became related to openness to experience (β = 0.111, *p* = 0.032). When adjusting for covariates, the PSS remained significantly associated with neuroticism (β = 0.255, *p* < 0.001), but was no longer related to extraversion (β = −0.041, *p* = 0.298), agreeableness (β = −0.076, *p* = 0.057), conscientiousness (β = −0.071, *p* < 0.076), or openness to experience (β = −0.050, *p* = 0.197).

In sum, all of the stress measures were weakly related to social desirability. In unadjusted analyses, the STRAIN showed fewer significant correlations with the personality factors measured than did the CTQ-SF and PSS. When controlling for relevant covariates, lifetime stressor count, as assessed by the STRAIN, performed similar to the CTQ-SF and worse than the PSS. On the other hand, lifetime stressor severity, as assessed by the STRAIN, performed better than both the CTQ-SF and PSS.

### Predictive Validity

The predictive validity of the STRAIN was evaluated in relation to six different health outcomes – namely, general mental health complaints (K-6), general physical health complaints (PHQ-BR), sleep quality (PSQI-BR), doctor-diagnosed general health problems, doctor-diagnosed autoimmune diseases, and a computer-based measure of executive function (Stroop). As hypothesized, greater lifetime stressor count, as assessed by the STRAIN, was significantly related to greater self-reported general mental health complaints (*r* = 0.444, *p* < 0.001) and physical health complaints (*r* = 0.367, *p* < 0.001), in addition to poorer sleep quality (*r* = 0.402, *p* < 0.001). Greater lifetime stressor count was also significantly related to having more doctor-diagnosed general health problems [Incidence Rate Ratio (IRR) = 1.031, 95% CI = 1.026–1.036, *p* < 0.001] and more doctor-diagnosed autoimmune disorders (IRR = 1.028, 95% CI = 1.008–1.048, *p* = 0.006). Contrary to expectations, lifetime stressor count was not related to participants’ Stroop interference scores (*r* = −0.016, *p* = 0.779).

Greater lifetime stressor severity, in turn, was also significantly associated with more self-reported general mental health complaints (*r* = 0.455, *p* < 0.001) and physical health complaints (*r* = 0.408, *p* < 0.001), as well as with poorer sleep quality (*r* = 0.418, *p* < 0.001). Greater lifetime stressor severity was also significantly related to having more doctor-diagnosed general health problems (IRR = 1.014, 95% CI = 1.011–1.016, *p* < 0.001) and more doctor-diagnosed autoimmune disorders (IRR = 1.011, 95% CI = 1.003–1.020, *p* = 0.011). As with lifetime stressor count, however, lifetime stressor severity was not related to participants’ Stroop interference scores (*r* = −0.036, *p* = 0.520).

When interpreting these results, we see that for each additional lifetime stressor detected by the STRAIN, participants were 3.1% more likely to have an additional doctor-diagnosed general health problem and 2.8% more likely to have an additional doctor-diagnosed autoimmune disorder. For each additional one-point increase in lifetime severity score (which can range from 1 to 5 for each stressor experienced), participants were 1.4% more likely to have an additional doctor-diagnosed general health problem and 1.1% more likely to have an additional doctor-diagnosed autoimmune disorder.

Importantly, all of these effects were robust while controlling for all of the covariates assessed (i.e., gender, age, race, socioeconomic status, and negative affect). Namely, greater lifetime stressor count remained significantly associated with more self-reported general mental health complaints (β = 0.278, *p* < 0.001) and physical health complaints (β = 0.268, *p* < 0.001), as well as with poorer sleep quality (β = 0.290, *p* < 0.001), more doctor-diagnosed general health problems (IRR = 1.030, 95% CI = 1.024–1.036, *p* < 0.001), and more doctor-diagnosed autoimmune disorders (IRR = 1.039, 95% CI = 1.014–1.065, *p* = 0.002). Additionally, lifetime stressor count continued to be unrelated to executive function as assessed by the Stroop (β = −0.037, *p* = 0.549). In the same way, greater lifetime stressor severity remained significantly associated with more self-reported general mental health complaints (β = 0.289, *p* < 0.001) and physical health complaints (β = 0.304, *p* < 0.001), as well as with poorer sleep quality (β = 0.305, *p* < 0.001), more doctor-diagnosed general health problems (β = 0.013, IRR = 1.013, 95% CI = 1.010–1.016, *p* < 0.001), and more doctor-diagnosed autoimmune disorders (β = 0.015, IRR = 1.015, 95% CI = 1.004–1.027, *p* = 0.008). Additionally, lifetime stressor severity also continued to be unrelated to executive function as assessed by the Stroop (β = −0.068, *p* = 0.283). Based on these results, we conclude that the Brazilian version of the STRAIN has excellent predictive validity, as evidenced by strong associations with all of the health outcomes evaluated except for executive function, both with and without adjustment for covariates.

### Comparative Predictive Validity

To examine the comparative predictive validity of the STRAIN relative to the other stress scales administered, we included each scale in the model simultaneously and adjusted for participants’ age, gender, race, socioeconomic status, and negative affect. As shown in Table 2, the STRAIN emerged as a significant predictor of five out of the six health outcomes assessed, which was substantially better than the CTQ-SF and slightly better than the PSS. In addition, the STRAIN was the only instrument that predicted doctor-diagnosed autoimmune disorders.

**TABLE 2:** Comparative predictive validity of the STRAIN, CTQ-SF, and PSS.

	**STRAIN**	**CTQ-SF**	**PSS**
	
**Health outcome**	**β**		
General mental health complaints (K-6)	0.164**	0.024	0.623**
General physical health complaints (PHQ-BR)	0.182*	0.063	0.323**
Sleep difficulties (PSQI-BR)	0.215**	0.019	0.401**
Executive dysfunction (Stroop)	−0.038	−0.060	0.205*
	**Incidence Rate Ratio [IRR]**		
Doctor-diagnosed general health problems	1.027**	1.002	1.017*
Doctor-diagnosed autoimmune disorders	1.037*	1.006	0.973

Significant values (*p* < 0.05) are presented in bold. All analyses included each stress scale simultaneously and adjusted for participants’ age, gender, race, socioeconomic status, and negative affect. Models with β coefficient indicate linear regression analysis, whereas models with Incidence Rate Ratio indicate Poisson regression analysis from generalized linear model. STRAIN, Stress and Adversity Inventory; CTQ-SF, Childhood Trauma Questionnaire-Short Form; PSS, Perceived Stress Scale; K-6, Kessler 6-Item Psychological Distress Inventory; PHQ-BR, Physical Health Questionnaire in Brazilian Portuguese; PSQI-BR, Brazilian Portuguese version of the Pittsburgh Sleep Quality Inventory. *p < 0.05; **p < 0.01.

Table 3 shows the percentage of variance explained by the STRAIN over and above the other stress scales administered in addition to participants’ age, gender, race, socioeconomic status, and negative affect. Notably, the STRAIN substantially increased the explanatory power of these models, especially with respect to predicting doctor-diagnosed general health problems (39.06% increase in variance explained) and autoimmune disorders (64.51% increase in variance explained), thus demonstrating the excellent incremental validity of the STRAIN.

**TABLE 3:** Incremental validity of the STRAIN over the CTQ-SF, PSS, and relevant covariates.

	**Health outcome**					
**Model**	**Mental health complaints (K-6)**	**Physical health complaints (PHQ-BR)**	**Sleep difficulties (PSQI-BR)**	**Executive dysfunction (Stroop)**	**Doctor-diagnosed general health problems**	**Doctor-diagnosed autoimmune disorders**
Covariates	Total *R*^2^: 0.459	Total *R*^2^: 0.248	Total *R*^2^: 0.196	Total *R*^2^: 0.005	Total *R*^2^: 0.176	Total *R*^2^: 0.020
Covariates + CTQ-SF +	Total *R*^2^: 0.684	Total *R*^2^: 0.340	Total *R*^2^: 0.311	Total *R*^2^: 0.025	Total *R*^2^: 0.256	Total *R*^2^: 0.031
PSS	Δ*R*^2^: 0.225	Δ*R*^2^: 0.092	Δ*R*^2^: 0.115	Δ*R*^2^: 0.020	Δ*R*^2^: 0.080	Δ*R*^2^: 0.011
Covariates + CTQ-SF + PSS + STRAIN	Total *R*^2^: 0.700 Δ*R*^2^: 0.016	Total *R*^2^: 0.359 Δ*R*^2^: 0.019	Total *R*^2^: 0.337 Δ*R*^2^: 0.026	Total *R*^2^: 0.025 Δ*R*^2^: 0.000	Total *R*^2^: 0.356 Δ*R*^2^: 0.100	Total *R*^2^: 0.051 Δ*R*^2^: 0.020

% of variance explained by the Brazilian STRAIN over and above the total variance previously explained	2.34%	5.59%	8.36%	0%	39.06%	64.51%

*STRAIN, Stress and Adversity Inventory; CTQ-SF, Childhood Trauma Questionnaire-Short Form; PSS, Perceived Stress Scale; K-6, Kessler 6-Item Psychological Distress Inventory; PHQ-BR, Physical Health Questionnaire in Brazilian Portuguese; PSQI-BR, Brazilian Portuguese version of the Pittsburgh Sleep Quality Inventory.*

### Test–Retest Reliability

Next, we examined the test–retest of the STRAIN for all participants who consented to receive a follow-up assessment (*n* = 270). In all, 79 participants completed the follow-up assessment after an average of 34.86 days (*SD* = 22.99; range: 14–96 days). Excellent reliability was observed for the two main STRAIN outcomes: total lifetime stressor count (*r*_icc_ = 0.936, *p* < 0.001; *r* = 0.881, *p* = ≤ 0.001) and total lifetime stressor severity (*r*_icc_ = 0.953, *p* < 0.001; *r* = 0.914, *p* < 0.001). Notably, both of these lifetime stress exposure indices require that participants accurately recall both the specific stressors that they experienced *and* their frequency across the entire lifespan, as well as the severity of such exposures in the case of the latter outcome. The Brazilian version of the STRAIN thus exhibits excellent reliability over time.

### Stress Exposure Characteristics

**FIGURE 2:**
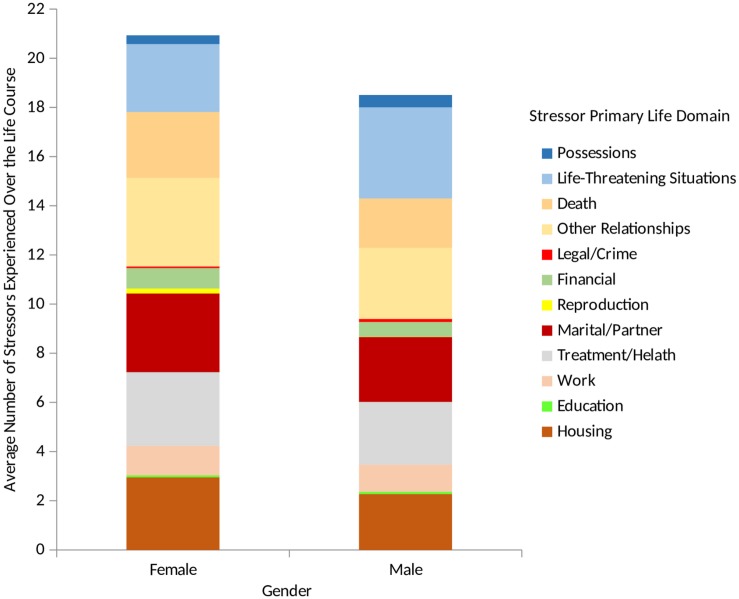
Lifetime stressor exposure by stressor count for males and females. Examining participants’ lifetime stress exposure by gender revealed that with respect to primary life domain, women experienced more reproduction stressors, other relationship stressors, and deaths than men. In contrast, men experienced more life-threatening stressors than women.

Due to the wealth of information provided by the STRAIN, we were able to describe differences in lifetime stressor exposure for men vs. women across the 12 primary life domains and five social-psychological characteristics assessed by the instrument. As shown in Figure 2, women experienced more stressors in the domains of reproduction (*M*_female_ = 0.20, *SD* = 0.53 vs. *M*_male_ = 0.01, *SD* = 0.11, *p* = 0.001, *d* = 0.49), other relationships (*M*_female_ = 3.61, *SD* = 3.04 vs. *M*_male_ = 2.89, *SD* = 2.61, *p* = 0.048, *d* = 0.25), and death (*M*_female_ = 2.68, *SD* = 1.99 vs. *M*_male_ = 2.01, *SD* = 1.89, *p* = 0.006, *d* = 0.34), whereas men reported more stressors involving life-threatening situations (*M*_female_ = 2.76, *SD* = 2.79 vs. *M*_male_ = 3.70, *SD* = 3.49, *p* = 0.011, *d* = 0.30).

**FIGURE 3:**
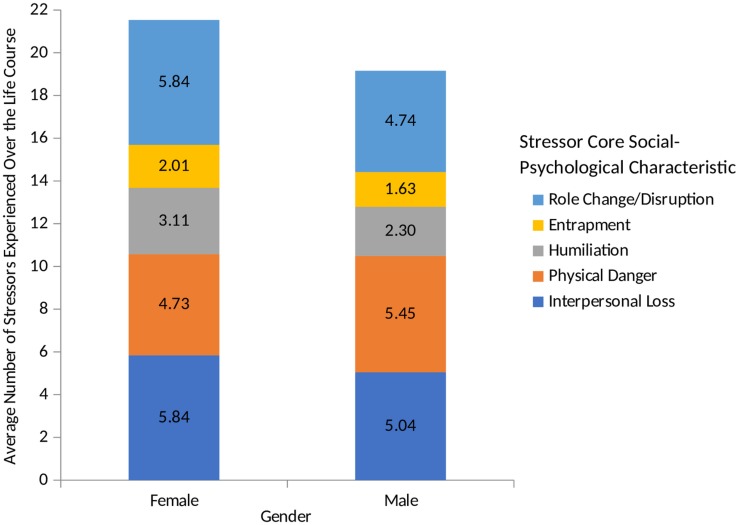
Lifetime stressor exposure by core social-psychological characteristics for males and females. Examining participants’ lifetime stress exposure by gender revealed that with respect to core social-psychological characteristic, women experienced more interpersonal loss, entrapment, humiliation, and role change/disruption stressors than men.

We conducted parallel analyses for the five core social-psychological characteristics assessed by the STRAIN. As shown in Figure 3, women reported significantly more stressors involving interpersonal loss (*M*_female_ = 5.84, *SD* = 3.14 vs. *M*_male_ = 5.04, *SD* = 3.11, *p* = 0.039, *d* = 0.26), entrapment (*M*_female_ = 2.01, *SD* = 1.51 vs. *M*_male_ = 1.63, *SD* = 1.344, *p* = 0.033, *d* = 0.27), humiliation (*M*_female_ = 3.11, *SD* = 2.91 vs. *M*_male_ = 2.30, *SD* = 2.42, *p* = 0.019, *d* = 0.30), and role change/disruption (*M*_female_ = 5.84, *SD* = 4.67 vs. *M*_male_ = 4.74, *SD* = 3.98, *p* = 0.048, *d* = 0.25) as compared to men.

**FIGURE 4:**
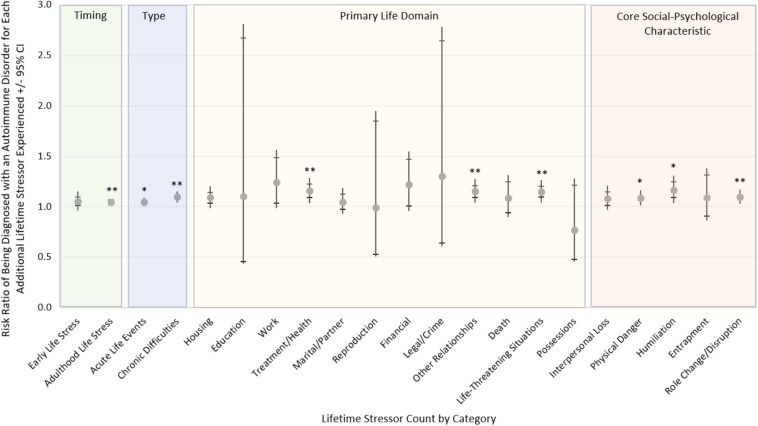
Likelihood of being diagnosed with an autoimmune disorder by stressor timing, type, primary domain, and core social-psychological characteristic. Risk of being diagnosed with an autoimmune disorder differed substantially across the different types of life stressors experienced, controlling for participants’ age, gender, race, socioeconomic status, and negative affect. More specifically, participants’ risk was greater for those experiencing adulthood vs. early life stressors and for those experiencing chronic vs. acute stressors. Risk was also greater for individuals experiencing stressors involving treatment/health, other relationships, life-threatening situations, physical danger, humiliation, and role change/disruption. ^∗^*p* < 0.05, ^∗∗^*p* < 0.01 (*n* = 330).

Finally, we examined the predictive validity of the different types of lifetime stress exposure assessed by the STRAIN in relation to participants’ likelihood of being diagnosed with an autoimmune disorder. This involved taking the cumulative lifetime stressor count variable and disaggregating it into exposure timing, stressor type, primary life domain, and core social-psychological characteristic. As shown in Figure 4, while controlling for participants’ age, gender, race, socioeconomic status, and negative affect, these stressor categories were not uniformly associated with participants’ risk of having a doctor-diagnosed autoimmune disorders. Rather, risk of being diagnosed with an autoimmune disorder was relatively greater for adulthood (vs. early life) stress exposure and for individuals experiencing chronic difficulties (vs. acute life events). Risk was also relatively greater for stressors involving treatment/health, other relationships, life-threatening situations, physical danger, humiliation, and role change/disruption.

## Discussion

Although many major contemporary theories of stress and health posit that stressors occurring over the entire lifespan can exert a cumulative effect on health, whereby stress burden and its negative effects increase over time, very few empirical studies have actually assessed individuals’ lifetime stress exposure given the difficulty of doing so (Shields and Slavich, 2017). In fact, a vast majority of studies on stress and health have utilized brief checklist measures that assess stress exposure occurring over a maximum of 1 week or month, leaving the remainder of the person’s life unexplored (Slavich, 2016a, 2019). We addressed this important issue in the present study by translating the Adult STRAIN into Brazilian Portuguese, with the ultimate goal of helping to extend high-quality stress assessment to Brazil.

Similar to the original Adult STRAIN, the Adult STRAIN in Brazilian Portuguese was completed relatively quickly (Brazilian Adult STRAIN: *M* = 16 min, 27 s vs. English Adult STRAIN: *M* = 18 min, 39 s). Likewise, there were no reported issues or complaints. The Brazilian Portuguese STRAIN demonstrated good concurrent validity with the CTQ-SF and PSS. As with the original Adult STRAIN, total lifetime stressor count as assessed by the Brazilian Portuguese STRAIN was significantly correlated with the CTQ-SF (Brazilian Adult STRAIN: *r* = 0.594; Adult STRAIN: *r* = 0.552) and the PSS (Brazilian Adult STRAIN: *r* = 0.377; Adult STRAIN: *r* = 0.147). Regarding the STRAIN’s discriminant validity, lifetime stressor count as assessed by the original Adult STRAIN was unrelated to personality characteristics or social desirability. In contrast, lifetime stressor count as assessed by the Brazilian Portuguese STRAIN was weakly but significantly related to both personality characteristics and social desirability. However, the Brazilian Portuguese STRAIN showed fewer significant correlations with the personality factors assessed, and lifetime stressor severity performed better than the CTQ-SF and PSS. Therefore, the STRAIN consistently exhibits better discriminant validity in general as compared to these other stress measures, although the findings involving the TIPI may be inconclusive given the relative brief nature of the TIPI and its resulting low internal consistency.

With respect to predictive validity, the STRAIN emerged as a significant predictor of five of the six health outcomes assessed, both with and without controlling for covariates. These outcomes included general physical and mental health complaints, sleep quality, doctor-diagnosed general health problems, and doctor-diagnosed autoimmune disorders. Additionally, the STRAIN was the only instrument that predicted number of doctor-diagnosed autoimmune disorders, which highlights its potential utility in the clinic where it could be used to assess psychosocial risk for these highly burdensome health problems. The predictive validity of the Brazilian Portuguese STRAIN was similar to that of the original Adult STRAIN validation study in English (Slavich and Shields, 2018) with the exception that in the original study, the STRAIN also significantly predicted executive function. Whereas the Brazilian Portuguese STRAIN significantly predicted five of the six health outcomes assessed, the original STRAIN significantly predicted all six of the health outcomes assessed – namely, general physical and mental health complaints, sleep quality, and executive function (βs ranged from 0.168 to 0.401), as well as doctor-diagnosed general health problems (risk ratio [RR] = 1.021) and doctor-diagnosed autoimmune disorders (RR = 1.038).

Finally, when we directly compared the STRAIN to the CTQ-SF and PSS in models that simultaneously adjusted for each of these instruments in addition to participants’ gender, age, race, socioeconomic status, and negative affect, the Brazilian Portuguese STRAIN explained a full 39.06% of the total explained variance in doctor-diagnosed general health problems and 64.51% of the total explained variance in doctor-diagnosed autoimmune disorders over and above the variance explained by the two other stress scales and all of the covariates assessed. This finding was similar though more impressive than that obtained in the original Adult STRAIN validation study (Slavich and Shields, 2018), wherein the STRAIN explained 30.42% of the total explained variance in doctor-diagnosed general health problems and 30.21% of the total explained variance in doctor-diagnosed autoimmune disorders over and above the variance explained by the two other stress scales administered and all of the covariates assessed in that study. The STRAIN in Brazilian Portuguese thus demonstrates excellent incremental validity that even outperforms the original Adult STRAIN in some respects.

Aside from predictive validity, a major issue with existing stress assessment instruments is that they do not yield consistent stress levels over time, even when retrospectively assessing the same time period. This is often thought to result from poor memory on the part of participants, but it can also be caused by overly general or imprecise stressor questions that lead individuals to produce different answers for the same questions over repeated assessments (Slavich and Shields, 2018). The STRAIN addresses this issue by including substantial contextual information in each item. As a result, the test–retest reliability for total lifetime stressor count as assessed by the STRAIN in the present study was *r*_icc_ = 0.936 and the test–retest reliability for total lifetime stressor severity was *r*_icc_ = 0.953 after an average test–retest period of approximately 1 month (*M* = 34.86 days). These test–retest reliability indices are higher than those obtained in the original Adult STRAIN validation study (Slavich and Shields, 2018), wherein the test–retest reliability for total lifetime stressor count was *r*_icc_ = 0.919 and the test–retest reliability for total lifetime stressor severity was *r*_icc_ = 0.904 after an average test–retest period of approximately 2 weeks (median = 13 days; range: 9–36 days). These excellent test–retest metrics are impressive given that they are based on participants having accurately recalled not just whether a particular stressor occurred but how many times it occurred and, in the case of total stressor severity, how impactful the stressor was for them. Some of the stressors reported could have occurred recently, but many would have occurred several years ago, including during childhood, thus providing strong evidence of the STRAIN’s use as a reliable instrument for assessing lifetime stress exposure.

Finally, based on prior studies showing that different stressors exert varying effects on health (e.g., Keller et al., 2007; Slavich et al., 2009, 2014; Murphy et al., 2013, 2015; Massing-Schaffer et al., 2019), we examined patterns of association between the different types of stress exposure assessed by the STRAIN and the various health outcomes measured. Women experienced more stressors in the life domains of reproduction, other relationships, and death, whereas men experienced more life-threatening stressors. This pattern was similar to that obtained in the original Adult STRAIN validation study (Slavich and Shields, 2018), with the exception that in the original study, women also experienced more treatment/health-related stressors whereas men experienced more legal/crime-related stressors. The present study thus obtained findings similar to the original validation study for women and different findings for men.

With respect to the core social-psychological characteristics assessed by the STRAIN, we found that women experienced more stressors involving interpersonal loss, humiliation, entrapment, and role change/disruption as compared to men, which is similar to what was previously reported for the English STRAIN (i.e., females were found to experience more interpersonal loss and entrapment stressors, and marginally more physical danger and humiliation stressors) (see Slavich and Shields, 2018). Finally, when we analyzed the predictive power of the STRAIN in relation to being diagnosed with an autoimmune disorder as a function of the specific timing of stress exposure, stressor type, primary life domain, and core social-psychological characteristic, we found that risk of being diagnosed with an autoimmune disorder was greater for participants experiencing adulthood vs. early life stress and chronic vs. acute life stress, as well as for those experiencing stressors involving treatment/health, other relationships, life-threatening situations, physical danger, humiliation, and role change/disruption. These findings are consistent with those obtained in the original Adult STRAIN validation study (Slavich and Shields, 2018), which found that the risk of being diagnosed with an autoimmune disorder was greater for those experiencing stressors in adulthood (relative to early life) and chronic stressors (relative to acute stressors). However, in the original validation study, autoimmune disorder diagnosis risk was most strongly associated with stressors involving possessions, reproduction, death, interpersonal loss, and physical danger.

Several limitations of this study should be noted. First, we conducted longitudinal analyses for evaluating the test–retest reliability of the STRAIN, but all other analyses were cross-sectional and based on constructs that were measured concurrently. Therefore, additional research using longitudinal study designs is needed to assess temporal precedence as well as prospective associations between life stress exposure and changes in health status over time. Second, since the STRAIN was weakly related to social desirability and to some personality traits, we cannot rule out the possibility that these processes could have influenced results involving self-reported outcomes. For instance, stress could impact personality (Shields et al., 2016). On the other hand, people with certain personality traits or dispositions may be more likely to under- or over-report certain stressors. Third, we did not assess biomarkers in the present study, highlighting the need to validate the Brazilian STRAIN against markers of disease risk that could not possibly be affected by self-reporting biases (e.g., cortisol, cytokine levels; Armer et al., 2018; Furman et al., 2019; Shields et al., 2019b; Slavich, 2020), as well as against other measures of stress exposure (e.g., investigator-based measures, speech samples, etc.; Slavich et al., 2019b). Finally, additional studies are needed to examine the generalizability of the present results in other more diverse samples, as well as in different clinical populations.

Notwithstanding these limitations, the present study is the first to validate the Adult STRAIN in Brazilian Portuguese and one of the first to systematically examine associations between lifetime stress exposure and a variety of health outcomes in any population. We found that the Adult STRAIN in Brazilian Portuguese exhibits excellent usability and acceptability, good concurrent and discriminant validity, consistent predictive validity across a variety of different outcomes, excellent incremental validity, and outstanding test–retest validity over an average of 1 month. The STRAIN is thus a highly practical, reliable, and valid instrument for assessing lifetime stress exposure in Brazilian Portuguese. Given the many stress exposure scores produced by the STRAIN, the instrument may be useful for researchers and clinicians who could benefit from obtaining a comprehensive picture of individuals’ exposure to stress across the life course.

## Data Availability Statement

All relevant data are within the manuscript and its Supporting Information files. See Supplementary Table 1 for all data.

## Ethics Statement

All study procedures were approved by the relevant Brazilian research bodies (i.e., Scientific Commission and the Ethics Committee in Research from the Pontifical Catholic University of Rio Grande do Sul) and adhered to Brazilian Resolution 466 of December 12, 2012, of the National Health Council of Brazil, Ministry of Health (CNS 46/12).

## Author Contributions

MC and GMS: conceptualization. MC and GSS: data curation and formal analysis. MC, MO, and GMS: funding acquisition. MC, GSS, and GMS: methodology. MC, MO, and CS: project administration. CS, GSS, and GMS: software. MO and GMS: supervision. MC and GSS: visualization. MC, CS, GSS, and GMS: writing – original draft. MC, MO, CS, GSS, and GMS: writing – review and editing.

## Conflict of Interest

The authors declare that the research was conducted in the absence of any commercial or financial relationships that could be construed as a potential conflict of interest.
